# KIF5A transports collagen vesicles of myofibroblasts during pleural fibrosis

**DOI:** 10.1038/s41598-017-04437-7

**Published:** 2017-07-04

**Authors:** Hirotoshi Kamata, Yoshikazu Tsukasaki, Tsuyoshi Sakai, Reiko Ikebe, Julia Wang, Ann Jeffers, Jake Boren, Shuzi Owens, Takahiro Suzuki, Masaaki Higashihara, Steven Idell, Torry A. Tucker, Mitsuo Ikebe

**Affiliations:** 1 Department of Cellular and Molecular Biology, University of Texas Health Science Center Northeast, 11937 US Highway 271, Tyler, Texas 75708-3154 USA; 2 Department of Hematology, Kitasato University School of Medicine, 1-15-1 Kitasato, Minami-ku, Sagamihara, Kanagawa 252-0374 Japan

## Abstract

Fibrosis involves the production of extracellular matrix proteins in tissues and is often preceded by injury or trauma. In pleural fibrosis excess collagen deposition results in pleural thickening, increased stiffness and impaired lung function. Myofibroblasts are responsible for increased collagen deposition, however the molecular mechanism of transportation of procollagen containing vesicles for secretion is unknown. Here, we studied the role of kinesin on collagen-1 (Col-1) containing vesicle transportation in human pleural mesothelial cells (HPMCs). Among a number of cargo transporting kinesins, KIF5A was notably upregulated during TGF-β induced mesothelial-mesenchymal transition (MesoMT). Using superresolution structured illumination microscopy and the DUO-Link technique, we found that KIF5A colocalized with Col-1 containing vesicles. KIF5A knock-down significantly reduced Col-1 secretion and attenuated TGF-β induced increment in Col-1 localization at cell peripheries. Live cell imaging revealed that GFP-KIF5A and mCherry-Col-1 containing vesicles moved together. Kymography showed that these molecules continuously move with a mean velocity of 0.56 μm/sec, suggesting that the movement is directional but not diffusion limited process. Moreover, KIF5A was notably upregulated along with Col-1 and α-smooth muscle actin in pleural thickening in the carbon-black bleomycin mouse model. These results support our hypothesis that KIF5A is responsible for collagen transportation and secretion from HPMCs.

## Introduction

It is well established that diverse pleural injuries can activate pleural mesothelial cells (PMCs) and that these PMCs play a critical role in the progression of pleural fibrosis with lung restriction, as occurs in fibrothorax^1^. In such circumstances, PMCs change their phenotype through the acquisition of mesenchymal properties (MesoMT) in response to mediators elaborated during pleural inflammation prone to fibrotic repair^2^. Based on the reports of our group and others^3–7^, it is thought that this process is critical to the pathogenesis of pleural organization and ultimately fibrosis *in vivo*.

PMCs can undergo a process termed mesomesenchymal transition (MesoMT) in response to stimulation by TGF-β and procoagulant or fibrinolytic proteases^3, 7, 8^. During the transition, mesothelial cells begin expressing α-SMA and demonstrate phenotypic changes. Transitioning cells also increase the expression and secretion of extracellular matrix (ECM) proteins such as Col-1, which is directly involved in pleural fibrosis.

However, the mechanism underlying this process is poorly understood. Our objective is to elucidate the mechanism of increased Col-1 secretion from HPMCs. We hypothesized that Col-1 containing vesicles are transported from the perinuclear domain of the cellular periphery through specific motor proteins rather than by simple diffusion. This process would facilitate efficient secretion from PMCs and the deposition of collagen fibers.

It is known that microtubule based motor systems primarily govern the directional transportation of intracellular vesicles from perinuclear region to cell peripheries^9–11^. In the case of melanocytes, melanosomes move from perinuclear domain to cell peripheries via the microtubule system using kinesin and dynein motors^12–15^. In contrast, the mechanism of Col-1 transportation and secretion and the identity of motor proteins responsible for these processes are unknown.

Kinesin is a microtubule based motor protein, which constitutes a super family of more than fifteen members and plays various roles in diverse cell motility and transportation events^16–18^. Among the kinesin family members, several kinesins are thought to be responsible for intracellular vesicular movements because of the processive nature of their movement^19–22^. These kinesins are kinesin 1 (KIF5A, B, and C), kinesin 2 (KIF3A,B, and C), KIF13A and B, KIFC2 and 3^10^. Based on their functions, we predicted that one of these kinesins would be capable of transporting and secreting vesicular Col-1.

In the present study, we sought to identify the transportation mechanism responsible for Col-1 secretion and deposition into the pleural space using advanced molecular and cellular imaging approaches. Using HPMCs, we found that KIF5A is a motor protein responsible for Col-1 transportation and secretion during MesoMT. It is expected that this work will shed new light on our understanding of the cellular derangements and mechanisms that contribute to pleural injuries and repair, thus providing clues that can be exploited to develop novel therapies for pleural fibrosis.

## Results

### KIF5A expression is enhanced during MesoMT of HPMCs with TGF-β stimulation

**Figure 1:**
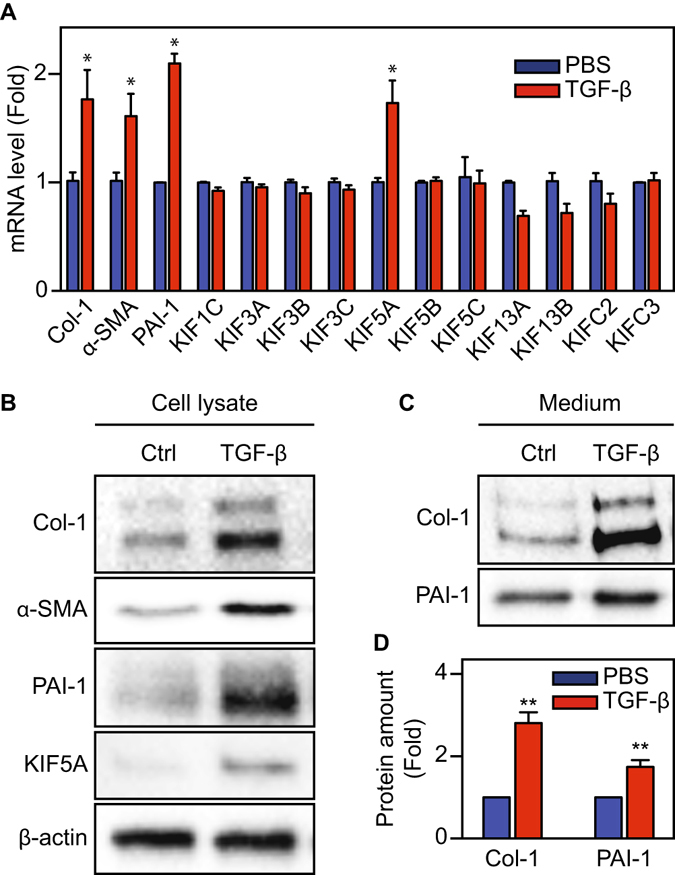
Upregulation of motor proteins during MesoMT. (**A**) mRNA level of motor proteins during MesoMT (n = 6). (**B**,**C**) Protein amount were investigated by western blotting in both cell lysate (**B**) and culture medium (**C**). Full-length blots were presented in Supplementary Figs S2 and S3. (**D**) Quantitative analysis using densitometry of western blotting of C (n = 3 for Col-1, n = 4 for PAI-1). *P < 0.05, **P < 0.01 vs basal level.

It was previously shown that HPMCs undergo MesoMT after TGF-β stimulation. Consequently, collagen secretion was enhanced in transitioning cells^23, 24^. We first studied what motor proteins can contribute collagen secretion. Serum starved HPMCs were stimulated with TGF-β for 24 hours. Total mRNA was isolated from the cells, transcribed into cDNA and then subjected to quantitative PCR analysis to determine changes in mRNA level. Among members of the kinesin superfamily, we examined isoforms that are suitable for cargo transport^16, 19^. KIF5A, specifically, was significantly upregulated after TGF-β stimulation (Fig. 1A). Western blot analyses also showed a marked increase in KIF5A protein expression after TGF-β stimulation (Fig. 1B). Markers of MesoMT α-SMA, PAI-1 and Col-1 (mRNA and protein) were likewise increased in the lysates of TGF-β treated cells (Fig. 1A,B)^7, 8, 23, 25^.

### Collagen secretion is induced during MesoMT of HPMCs stimulated by TGF-β

It is anticipated that the increased transportation of Col-1 containing vesicles facilitates Col-1 secretion. To address this point, we measured secreted proteins in HPMC conditioned culture medium by western blot analysis. Col-1 and PAI-1 levels in the culture media were significantly increased during TGF-β induced MesoMT (Fig. 1C,D). Further, we found a notable increase of paracellular collagen fiber formation after TGF-β stimulation (Supplementary Fig. S1A). These results suggest that TGF-β stimulation not only increases RNA and protein expression of Col-1 but also enhances Col-1 secretion from HPMCs.

### TGF-β induces Col-1 vesicle localization towards cell peripheries

**Figure 2:**
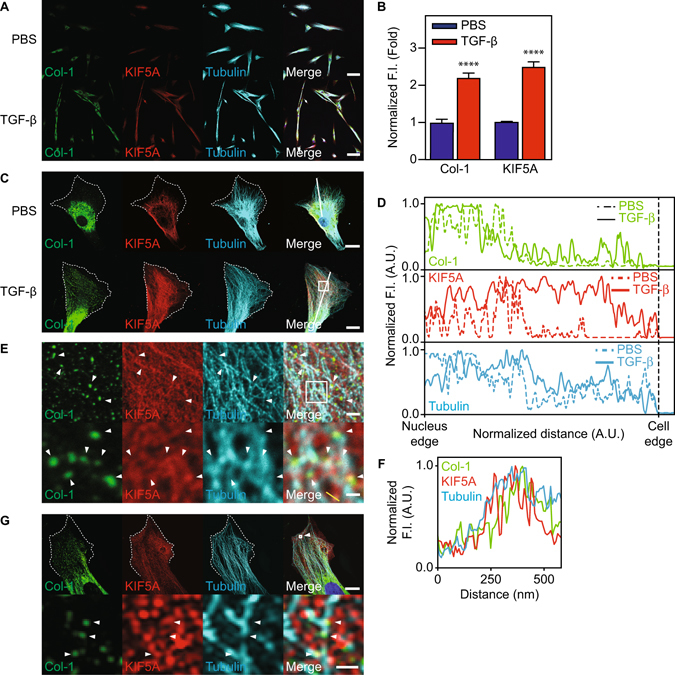
Effect of TGF-β on localization of KIF5A and Col-1 in HPMCs. (**A**) Confocal images of multiple HPMCs with or without TGF-β stimulation. Merge images include blue nucleus signal. Scale bar; 60 μm. (**B**) Quantitative analysis of average fluorescent intensity inside the cells of A (n = 8 cells for PBS, n = 9 cells for TGF-β). The intensity was normalized with the control. ****P < 0.0001 vs basal level. F.I., Fluorescent Intensity. (**C**) Confocal images of single HPMC with or without TGF-β stimulation. Merge images include blue nucleus signal. White dotted lines indicate the edge of cells. Scale bar; 20 μm. (**D**) Quantitative line profile along white lines of C. F.I., Fluorescent Intensity. (**E**) Magnified images of intracellular structures. White box in C was magnified. White box in upper image was magnified to lower images. White arrows indicate colocalization among Col-1 and KIF5A and tubulin. Scale bar; 2 μm in upper image, 0.5 μm in lower image. (**F**) Quantitative line profile of co-localization along yellow lines of E. F.I., Fluorescent Intensity. (**G**) Superresolution images of single HPMC with TGF-β stimulation. Merge images include blue nucleus signal. White box with arrow in upper image was magnified to lower images. White dotted lines indicate the edge of cells. Scale bar; 10 μm in upper image, 0.5 μm in lower image.

**Figure 3:**
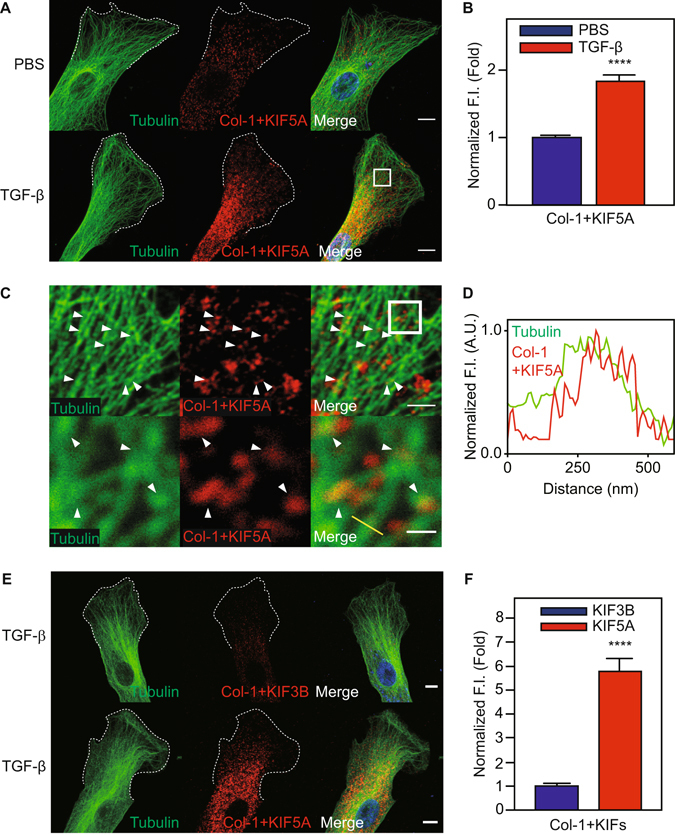
Proximity-ligation assay for colocalization of KIF5A and Col-1 in HPMC. (**A**) Confocal images of single HPMCs with proximity-ligation assay with or without TGF-β stimulation. Merge images include blue nucleus signal. White dotted lines indicate the edge of cells. Scale bar; 10 μm. (**B**) Quantitative analysis of average fluorescent intensity inside the cells of A (n = 6 cells). ****P < 0.0001 vs basal level. F.I., Fluorescent Intensity. (**C**) Magnified images of intracellular structures. White box in A was magnified. White box in upper image was magnified to lower images. White arrows indicate colocalization among Col-1 and KIF5A and tubulin. Scale bar; 2 μm in upper image, 0.5 μm in lower image. (**D**) Quantitative line profile of co-localization along the yellow line of C. F.I., Fluorescent Intensity. (**E**) Confocal images of single HPMCs with proximity-ligation assay of KIF3B or KIF5A and Col-1 with TGF-β stimulation. Merge images include blue nucleus signal. White dotted lines indicate the edge of cells. Scale bar; 10 μm. (**F**) Quantitative analysis of average fluorescent intensity of proximity-ligation signal inside the cells of E (n = 10 cells). Fluorescent intensity of KIF3B and Col-1 was taken as 1. ****P < 0.0001 vs basal level. F.I., Fluorescent Intensity.

Because TGF-β induced MesoMT increases Col-1 secretion, we next studied TGF-β mediated changes in the intracellular localization of Col-1 containing vesicles. Col-1 vesicles were concentrated at the perinuclear region of untreated HPMCs. After TGF-β treatment, these vesicles became distributed towards the cell peripheries (Fig. 2A–D). Furthermore, KIF5A expression was enhanced by treatment with TGF-β (Fig. 2A–D). These results indicate that TGF-β not only increases the total expression of Col-1 but also changes distribution of the Col-1 containing vesicles in HPMCs. KIF5A localization at cell peripheries was likewise increased after TGF-β stimulation. These results support our assertion that the KIF5A transport system is responsible for the mobilization of Col-1 containing vesicles from the perinuclear region to cell peripheries. It should be noted that we can not exclude the possibility that the change in the microtubule structure may be in part involved in the distribution of the Col-1 containing vesicles, since there is some change in microtubule around the cell periphery after TGF-β stimulation (Figs 2C and 3A).

### KIF5A and Col-1 colocalize on tubulin fibers in HPMCs

Because Col-1 and KIF5A localized to the cell periphery, we next examined if these proteins colocalized in TGF-β treated cells. We analyzed the colocalization of Col-1 containing vesicles, KIF5A and microtubules in HPMCs by staining with specific antibodies using Laser Scanning Confocal microscopy (Fig. 2E,F) and super resolution SIM (Fig. 2G). We observed KIF5A and Col-1, respectively, colocalized on tubulin fibers in TGF-β treated HPMC (Fig. 2E–G). However, a notable fraction of these molecules were found not to be associated with microtubules (Fig. 2E–G). We also found that KIF5A colocalized with Col-1 in TGF-β treated cells (Fig. 2E–G). Further, colocalized KIF5A/Col-1 was found on microtubules (Fig. 2E,G, white arrowheads and yellow line). To determine the relative proximity of KIF5A and Col-1 containing vesicles, we performed a proximity-ligation assay^26, 27^, which enables us to quantitatively detect protein-protein interactions within 30–40 nm with high specificity and sensitivity. Using this recently developed technique, we found that TGF-β stimulation markedly increased fluorescent signal of proximity-ligation assay between KIF5A and Col-1 containing vesicles (Fig. 3A,B). On the other hand, the proximity-ligation signal between KIF3B and Col-1 was much lower than that between KIF5A and Col-I. (Fig. 3E,F). The results suggest that the interaction between KIF5A and Col-1 is specific. Moreover, we found that proximity-ligation signals of KIF5A/Col-1 interaction reside on microtubules (Fig. 3C,D, white arrowheads and yellow line). These results suggest that KIF5A associates with the Col-1 containing vesicles and is likely responsible for the transportation of Col-1 containing vesicles in HPMCs.

### KIF5A down-regulation decreases collagen secretion

**Figure 4:**
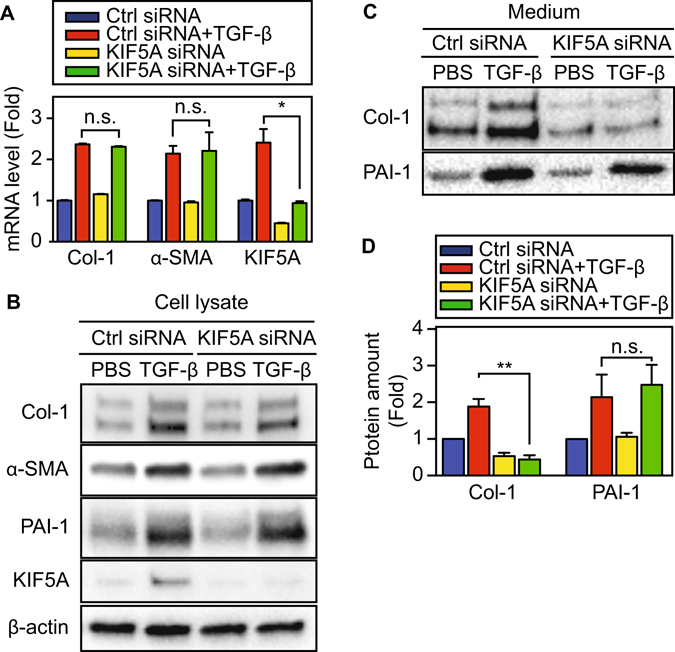
Effect of KIF5A KD on collagen secretion from HPMC. (**A**) Effect of KIF5A KD on mRNA level (n = 6). (**B**,**C**) Effect of KIF5A KD on protein amount in cell lysate (**B**) and in culture medium (**C**). Full-length blots were presented in Supplementary Figs S4 and S5. (**D**) Quantitative analysis using densitometry of western blotting of C (n = 3 for Col-1, n = 4 for PAI-1). *P < 0.05, **P < 0.01 vs basal level.

**Figure 5:**
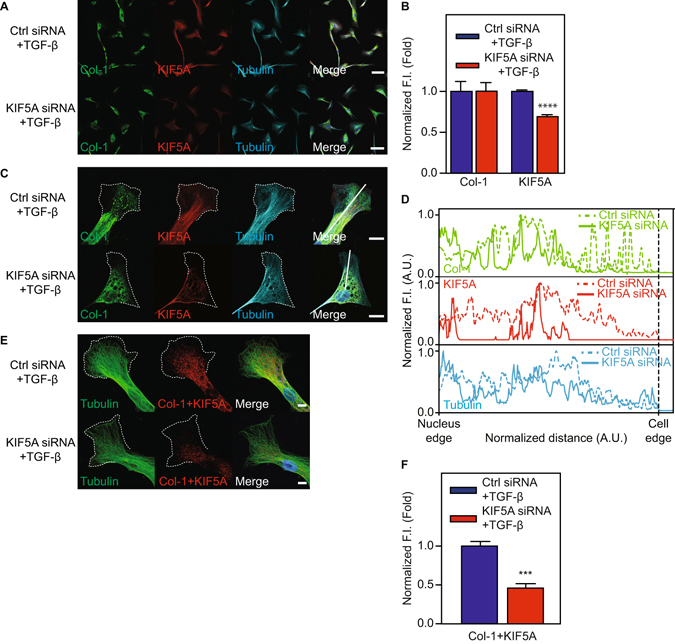
Effect of KIF5A KD on collagen transportation in HPMC. (**A**) Confocal images of multiple HPMCs treated with control siRNA and KIF5A siRNA after TGF-β stimulation. Merge images include blue nucleus signal. Scale bar; 60 μm. (**B**) Quantitative analysis of average fluorescent intensity inside the cells of A (n = 11 cells for control siRNA, n = 9 cells for KIF5A). ****P < 0.0001 vs basal level. F.I., Fluorescent Intensity. (**C**) Confocal images of single HPMC treated with control siRNA and KIF5A siRNA after TGF-β stimulation. Merge images include blue nucleus signal. White dotted lines indicate the edge of cells. Scale bar; 20 μm. (**D**) Quantitative line profile along white lines of C. F.I., Fluorescent Intensity. (**E**) Confocal images with proximity-ligation assay of single HPMCs treated with control siRNA and KIF5A siRNA after TGF-β stimulation. White dotted lines indicate the edge of cells. Scale bar; 10 μm. (**F**) Quantitative analysis of average fluorescent intensity inside the cells of E (n = 4 cells). ****P < 0.0001 vs basal level. F.I., Fluorescent Intensity.

Although we showed that KIF5A and Col-1 vesicles colocalize in TGF-β treated cells, the role of KIF5A in Col-1 transport was not clear. To further determine the role of KIF5A in Col-1 transport, we down-regulated KIF5A expression with targeting siRNA and monitored Col-1 transportation and secretion. Quantitative PCR analysis revealed that KIF5A siRNA significantly reduced the KIF5A mRNA level both before and after TGF-β stimulation (KD efficiency; 55% for before, 61% for after) (Fig. 4A), while the expression of Col-1 and α-SMA was unchanged. Furthermore, TGF-β induced increases in KIF5A protein expression was abolished by KIF5A siRNA (Fig. 4B). Col-1, α-SMA and PAI-1 expression levels in the cellular lysates were not affected by KIF5A KD (Fig. 4B). We next determined whether KIF5A KD attenuates Col-1 secretion from HPMC. As shown in Fig. 4C and D, Col-1 secreted into conditioned culture medium was significantly reduced (p < 0.01) by KIF5A KD. Consistently, the TGF-β induced increase in paracellular collagen fiber formation was abolished (Supplementary Fig. S1B). PAI-1 secretion was unaffected by KIF5A KD. These results support that KIF5A is critical for the transport of Col-1 containing vesicles. Further, cell imaging analysis show that KIF5A siRNA markedly diminished KIF5A expression as well as TGF-β induced Col-1 localization at cell peripheries (Fig. 5A–D). Using a proximity-ligation assay^26, 27^, we also found that KIF5A KD markedly decreased the interaction between KIF5A and Col-1 containing vesicles (Fig. 5E,F). These results support our hypothesis that KIF5A transports Col-1 containing vesicles from perinuclear region to cell peripheries using microtubules.

### KIF5A transports col-1 containing vesicles in live HPMCs

**Figure 6:**
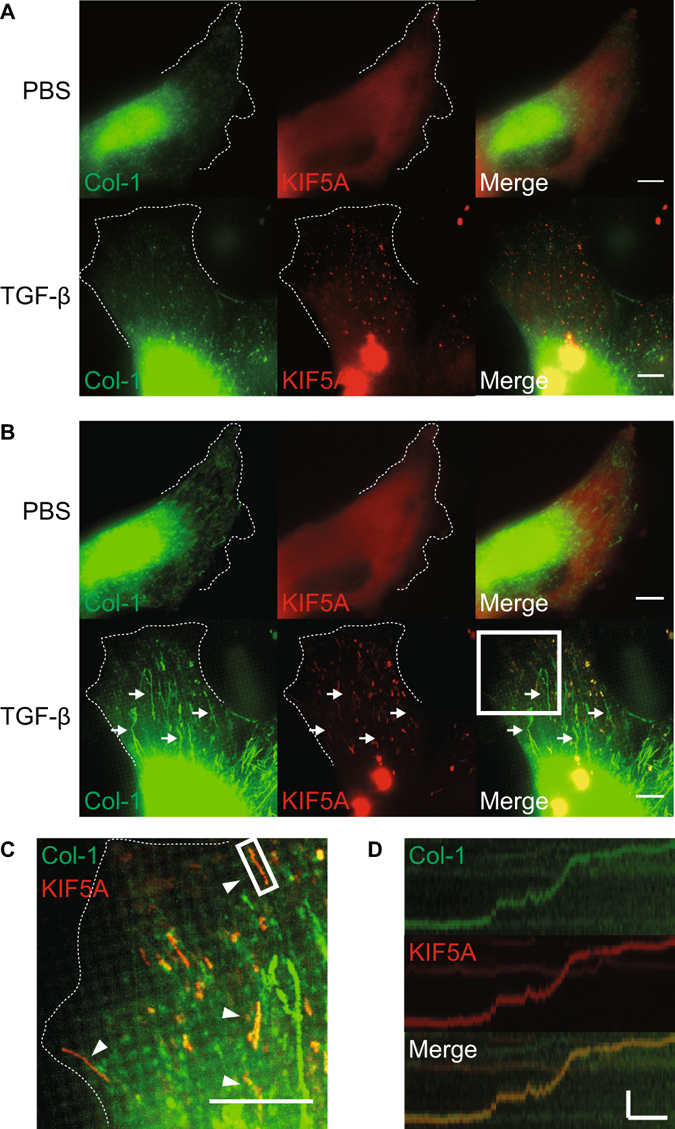
Live cell imaging of collagen transpotation in HPMC. (**A**) Snap-shot images of fluorescent protein labelled Col-1 and KIF5A with or without TGF-β stimulation in live HPMC. White dotted lines indicate the edge of cells. Colors are pseudocolors. Scale bar; 10 μm. (**B**) Time projection images of fluorescent protein labelled Col-1 and KIF5A with or without TGF-β stimulation in live HPMC. White dotted lines indicate the edge of cells. Colors are pseudocolors. Arrows indicate long distance movement of collagen vesicle. Scale bar; 10 μm. (**C**) Magnified time projection image. White box in B was magnified. Arrows indicate co-movement of Col-1 and KIF5A. White dotted lines indicate the edge of cells. Scale bar; 10 μm. (**D**) Kymograph analysis. Kymograph was generated from the location of white box in c. Colors are pseudocolors. Scale bar; 2 μm (horizontal bar) and 10 sec (vertical bar).

To obtain conclusive evidence that KIF5A transports Col-1 containing vesicles, we performed live cell imaging. HPMCs were co-transduced with mCherry-Col-1 expressing viral vector and GFP-KIF5A expressing viral vector. In live control PBS treated cells, GFP-KIF5A showed diffuse localization, while GFP-KIF5A showed punctate localization after TGF-β stimulation (Fig. 6A). The result suggests that TGF-β stimulation facilitates KIF5A association with Col-1 containing vesicles. Time projection images revealed that TGF-β stimulation notably facilitated the directional movement of Col-1 containing vesicles in live HPMCs (Fig. 6A and B). The trajectory images of mCherry-Col-1 and GFP-KIF5A, where the maximum intensity of each pixel during 30 seconds movie was projected, clearly showed the synchronized movement of mCherry-Col-1 and GFP-KIF5A (Fig. 6B and Supplementary Video 1). 62% of collagen vesicle co-localized with KIF5A signal and 81% of co-localized signal co-migrated (n = 114). The Kymograph analysis showing the time course of the directional movement of GFP-KIF5A and mCherry-Col-1 suggests that mCherry-Col-1 and GFP-KIF5A continuously move without dissociation from the track. The complex moved with a velocity of 0.56 ± 0.33 μm/sec (mean ± S.E., n = 54), stopped, and then restarted to move in the same direction towards cell peripheries (Fig. 6C). It should be noted that we also found relatively long distance of Col-1 movement with little GFP signal intensity (Fig. 6A Arrow) (see Discussion). In addition to transportation of Col-1 containing spherical vesicles, we also found the movement of large structure containing filamentous Col-1 near the plasma membrane (Supplementary Video 2). The results suggest that Col-1 (pro-collagen) containing vesicles fuse to form a large sac like vesicles near the plasma membrane, forming large fibers prior to exocytosis^28^.

### KIF5A expression is increased in the thickened visceral pleura of carbon black bleomycin injured mice

**Figure 7:**
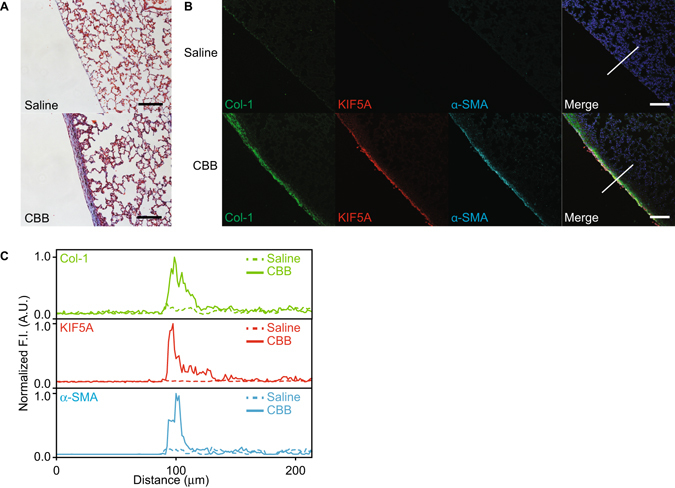
Mice pleural tissue of CBB-treated disease model. (**A**) Trichrome staining of pleural tissue of mice with or without CBB treatment. Scale bar; 100 μm. (**B**) Immuno-fluorescent staining of pleural tissue of mice with or without CBB treatment. Merge images include blue nucleus signal. Scale bar; 100 μm. (**C**) Quantitative line profile along white lines of B. F.I., Fluorescent Intensity.

Because we found that KIF5A was increased in HPMCs undergoing MesoMT we next labeled KIF5A in our previously published mouse model of pleural injury^7^. A critical question is whether or not KIF5A expression is upregulated during pleural fibrosis *in vivo*. We created fibrotic pleura using carbon black bleomycin (CBB) as was reported^23, 25^. We performed immunohistochemistry on the pleural tissue of carbon black bleomycin (CBB) treated mice. Trichrome staining for CBB treated tissue showed pleural thickening and collagen deposition in pleura (Fig. 7A). Immunofluorescent staining revealed marked increase in expression of KIF5A in pleural layer that colocalized with α-SMA, a marker of MesoMT. This coincided with marked increase in surrounding deposition of Col-1 (Fig. 7B,C). These results suggest that KIF5A expression is upregulated in pleural fibrotic tissues *in vivo*.

## Discussion

It is known that protocollagen is synthesized and post-translationally modified in the ER and then transported to the Golgi complex^29–32^. While a number of studies have been conducted to elucidate the role of various components in collagen secretory pathways including coat protein II complex (COPII)^33^, cytoplasmic GTPase^34^ and its regulatory proteins, little is known about the motor proteins that move collagen (procollagen) containing vesicles. The present study is the first to identify KIF5A as a motor protein that transports Col-1 containing vesicles in HPMCs. An earlier study reported that disruption of microtubules by Colchicine disrupted collagen secretion from cultured fibroblasts, suggesting the involvement of microtubule based transportation system for collagen secretion^35^. The present study is consistent with this earlier report, and identifies microtubule based motor protein KIF5A as responsible for the movement of Col-1 containing vesicles in cells that facilitates Col-1 secretion. This conclusion is based upon the following findings: (1) Among cargo transporting KIFs, KIF5A expression is notably upregulated during TGF-β induced activation of Col-1 secretion. (2) Col-1 and KIF5A show notable colocalization in HPMCs. (3) KIF5A KD by specific siRNA significantly decreases Col-1 secretion. (4) KIF5A KD abolished TGF-β induced Col-1 localization at cell peripheries, suggesting that KIF5A is responsible for Col-1 transportation. (5) Live cell imaging revealed KIF5A and Col-1 containing vesicles show continuous directional co-movement.

As described above, KIF5A KD limited the localization of Col-1 containing vesicles to the perinuclear region of the cell. It is known that microtubules extend from near the nucleus to cell peripheries with plus ends towards cell peripheries^20, 21^. Intracellular vesicles contain different motor proteins such as dynein and kinesin^36, 37^. Since KIF5A and dynein are plus and minus directed motors, respectively, it is anticipated that minus directed transportation by dynein motor dominates over KIF5A driven plus directed movement in KIF5A KD cells. Supporting this view, it was reported previously that KIF5B is responsible for endosomal vesicle transportation between the trans-Golgi network and perinuclear endosomal compartments. Further, the depletion of KIF5B resulted in accumulation of endosomal vesicles at the perinuclear region^38^.

Kinesin 1 family members including KIF5 form a folded and an extended conformation^39, 40^. In the folded conformation, the tail domain associates with the motor domain, inhibiting the ATP hydrolysis cycle and microtubule binding^41–44^. This tail-dependent inhibited conformation is altered to an active extended conformation when the kinesin binds to its target proteins^39, 45, 46^. Therefore, it is thought that the active form of KIF5A, but not its inactive folded form, can associate with microtubules to move with its cargo molecules. We found that KIF5A in live HPMCs showed diffuse localization before TGF-β stimulation. It is plausible that KIF5A is in an inactive conformation before TGF-β stimulation, thus it does not associate with the Col-1 containing vesicles. It should be noted that diffuse localization in the absence of TGF-β was not apparent in the fixed cells. We think that this is due to the loss of soluble KIF5A during the detergent based permeabilization of the cells. In the present study, we found notable colocalization and close association of KIF5A and Col-1 containing vesicles (Figs 2 and 3); however, a significant fraction of KIF5A was found to be without colocalizing with Col-1. These results suggest that a significant fraction of KIF5A in cells is in an inhibited conformation that is not associated with its cargo. Among the colocalized KIF5A-Col-1 vesicles, we found that a certain population of the complexes colocalized with microtubules, but not all of them. It is known that kinesin motor proteins such as KIF5A travel along microtubules for a limited distance, but they also dissociate from microtubules with a certain frequency after running for a certain distance^47^. Therefore, it is expected that the KIF5A/Col-1 complex is also present off microtubules. We also found relatively long distance movement of Col-1 containing vesicles with little GFP-KIF5A signal intensities. It has been thought that only a few motor molecules are sufficient to support intracellular cargo movement^48^. Therefore, it is likely that this movement is driven by low number of KIF5A molecules which do not provide enough detectable fluorescence intensities. Since excess motor molecules rather reduce the run-length due to non-harmonized interaction among the multiple motor molecules on microtubules^48^, it is plausible that the observed long distance movement of Col-1 containing vesicles is achieved by low number of KIF5A molecules. However, we cannot exclude the possibility that other kinesin motors are responsible for this movement.

The velocity of Col-1 containing vesicle movement operated by KIF5A was approximately 0.56 μm/sec. This value is a little slower than that obtained for *in vitro* single molecule assay (0.79 μm/sec)^49^. A possible explanation is the different environment of motor proteins between in cell and in *in vitro* experiments. Another explanation is that the tug of war between KIF5A and dynein causes a decrease in the plus directed movement of Col-1 containing vesicles, since the vesicles are likely to contain other motors than KIF5A. Such motors may include dynein, a minus directed microtubule based motor. The observation that KIF5A KD shifted the localization of Col-1 containing vesicles towards the perinuclear region supports our assertion that the vesicles contain both plus and minus directed motors.

While KIF5A is an important motor for Col-1 transportation and secretion, it is highly likely that additional motor proteins, such as myosins, are involved in the secretion, especially near plasma membrane for short distance movement, membrane fusion and secretion. It is reported for melanocytes that both kinesin and myosin Va are involved in the transportation and secretion of melanosomes^50–52^. Identification of myosin motors responsible for Col-1 secretion requires further study. It has been shown that KIF5A helps the association between microtubules and smad2, a down-stream effector of TGF-β, and this association affected the reorganization of cytoskeletons and TGF-β signaling^53, 54^. It is plausible that KIF5A is involved in signaling process and endosomal pathway via cytoskeletal rearrangements in addition to transportation of collagen vesicles. Further study on KIF5A functions for collagen secretion is needed to address this point.

Our results indicate that KIF5A is responsible for Col-1 transportation and secretion from transitioned HPMCs. As collagen secretion is found in a variety of cell types, further study will be required to determine if KIF5A is a common motor for collagen transportation in diverse cell types.

## Materials and Methods

### HPMC isolation and culture

HPMCs were collected in a deidentified manner and cultured from the pleural effusions of patients with congestive heart failure or who have undergone coronary artery bypass graft surgery, which would have otherwise been discarded^55^. All samples are collected in a deidentified manner through an exempt protocol approved by the Institutional Human Subjects Review Board of the University of Texas Health Science Center at Tyler (15-004). All experiments using these cells were performed in accordance with relevant guidelines and regulations. The cells were maintained on dishes with CellBIND surface (Corning) using LHC-8 culture medium (Gibco) containing 3% fetal bovine serum (Invitrogen), 2% antibiotic-antimycotic (Invitrogen), and 1% L-glutamine (Invitrogen) in a humidified incubator at 37 °C and 5% CO_2_/95% air.

### Cellular treatment

Cells were incubated in serum-free medium (SFM) of RPMI 1640 (Hyclone) with GlutaMAX supplement (Gibco) for 8–16 hours prior to recombinant human TGF-β (R&D systems) treatments. Serum-starved cells were treated with PBS or 5 ng/mL TGF-β in SFM. Cells were then allowed to incubate for 24 hours (Quantitative PCR analysis) or 48 hours (western blotting and immunostaining analysis) at 37 °C and 5% CO_2_/95% air.

### Quantitative PCR

Total mRNA was isolated using RNeasy Mini kit (Qiagen) according to the manufacturer’s instructions. Total mRNA was then reverse-transcribed into total cDNA using the SuperScript VILO (Invitrogen) according to the manufacturer’s instructions. Quantitative PCR analysis was then performed on the cDNA using QuantStudio 6 Flex (Applied Biosystems) and Taqman Assays for Collagen-I (Hs00164004_m1), KIFs (Hs00192120_m1, Hs00189659_m1, Hs00189672_m1, Hs00199901_m1, Hs01122781_m1, Hs00158482_m1, Hs00223154_m1, Hs00209573_m1, Hs01034147_m1, Hs00377525_m1, Hs00194304_m1), PAI-1 (Hs01126606_m1) and α-SMA (Hs00426835_g1) (Applied Biosystems). GAPDH (Applied Biosystems) was used as a loading control.

### Western blotting

Conditioned media (CM) was collected and cells were washed with PBS and lysed with 100 μl PBS containing 1% Triton-X 100 supplemented with Halt Protease Inhibitor Cocktail (ThermoFisher Scientific). Cell lysates were then incubated on ice for 30 min, and debris was removed by centrifuging. Protein concentrations of cell lysates were determined using Pierce BCA assay kit (ThermoFisher Scientific). CM and cell lysates were resolved by SDS-PAGE 4–20% polyacrylamide gradient gel (Bio-Rad) or 5% polyacrylamide gel, and α-SMA (1A4, R&D systems), Col-1 (1310-01, SouthernBiotech), PAI-1 (PAI3C311, Molecular Innovations), KIF5A (ab5628, Abcam), and β-actin (AC-15, Sigma Aldrich) antibodies were used for detection as described previously^56, 57^. Immunoblots were then imaged by Molecular Imager ChemiDoc XRS+ (Bio-Rad).

### Immunofluorescence staining

Cells were washed with PBS, placed in fixation solution (4% formaldehyde, 2 mM MgCl_2_, and 1 mM EGTA in PBS), washed with PBS, and permeabilized with 0.1% Triton X-100 in PBS for 10 min. Cells were then washed and blocked with 1% BSA including 0.02% azide in PBS for 30 min. Col-1 (1310-01, SouthernBiotech), KIF5A (ab118534, Abcam), KIF3B (A007119, Sigma) and tubulin (T4026, Sigma-Aldrich) antibodies were diluted with 1% BSA including 0.02% azide, then applied to cells and incubated overnight. First antibodies were visualized with Alexa Fluor 488, 568 and 647 secondary antibodies (Lifetechnologies), and nuclei were stained with Hoechst 33342 (ThermoFisher Scientific). Proximity-ligation assay was performed according to the Olink Bioscience protocol using Duolink *In situ* Red starter kit. After incubation with primary antibodies as described above, cells were washed and incubated with PLA probes (anti-goat MINUS and anti-rabbit PLUS) for 1 hour at 37 °C. Subsequent ligation and detection were performed according to the manufacturer’s protocol. Cells were mounted onto slides with ProLong Gold Antifade Reagent (Lifetechnologies). Fluorescence images were taken by Leica TCS SP8 systems (Leica Microsystems) for confocal microscopy and DeltaVision OMX (GE Healthcare Life Sciences) for superresolution SIM. Fluorescent intensity analysis and line profile analysis were performed by image J.

### KD experiment

Cells were transfected with ON-TARGET plus KIF5A siRNA SMART pool (Dharmacon) or control siRNA (Eurofins) for 4.5 hours at 37 °C in SFM using Lipofectamin 2000 (Invitrogen), then were maintained in LHC8 complete medium and used in the following experiments.

### Live cell imaging

GFP-KIF5A was cloned in BacMam pCMV-DEST Vector using the Gateway cloning system. The GFP-KIF5A expression virus (Baculovirus) in SF9 cells was created using ViraPower BacMam Expression System (ThermoFisher Scientific). The mCherry-Col-1 expression virus (Adenovirus) was purchased from Cyagen. Dual color live cell imaging was performed by using a DeltaVision OMX (GE Healthcare Life Sciences) structured illumination super resolution microscope (SIM) 48 hours after transfection with 5% CO2 and 37 °C. Line profile analysis and kymorgraph anaysis were performed using image J.

### CBB treatment of mice

All experiments involving animals were approved by the Institutional Animal Care and Use Committee at the University of Texas Health Science Center at Tyler. All experiments regarding to animals were performed in accordance with relevant guidelines and regulations. Wild-type C57BL/6j mice were treated with carbon black/bleomycin (CBB) or saline for 14 days as previously described^7^.

### Histochemistry and immunofluorescence staining of mice tissues

Tissues sections were first deparaffinized and subjected to antigen retrieval using a citrate buffer at 95 °C for 20 minutes as previously described^7^. Tissue morphology was next assessed by hematoxylin and eosin (H&E) staining as previously described^7^. Collagen deposition and localization were visualized by Trichrome as previously described^7^. Immunostaining was performed by using antibodies of Col-1 (1310-01, SouthernBiotech), α-SMA (1A4, R&D systems) and KIF5A (ab118534, Abcam) as previously described^7^. Briefly, mouse tissue sections were blocked using a proprietary blocking solution from a M.O.M. kit (Vector Laboratories). Primary antibodies were then incubated overnight at 4 °C in kit diluent. First antibodies were visualized with Alexa Fluor 488, 568, and 647 secondary antibody (Liftechnologies), and nuclei were stained with Hoechst 33342 (ThermoFisher Scientific). Tissues were mounted onto slides with ProLong Gold Antifade Reagent (Lifetechnologies). Tissue staining images were taken by ECLIPSE Ti (Nikon) for Trichrome staining and Leica TCS SP8 systems (Leica Microsystems) for immunofluorescence staining.

## Electronic supplementary material

Supplementary materials

Dual color live cell imaging of Col-1 (Green) and KIF5A (Red)

Movements of filamentous Col-1 (Green)
