# Supplementary material for: KIF5A transports collagen vesicles of myofibroblasts during pleural fibrosis
Source: Sci Rep. 2017 Jul 4;7:4556. doi: 10.1038/s41598-017-04437-7 (PMC5496869; doi:10.1038/s41598-017-04437-7)
Supplement: Supplementary file 1 — Supplementary materials [file 41598_2017_4437_MOESM1_ESM.pdf]

KIF5A transports collagen vesicles of myofibroblasts during pleural fibrosis

Hirotoshi Kamata[1, 2, 3], Yoshikazu Tsukasaki[1, 3], Tsuyoshi Sakai[1], Reiko Ikebe[1], Julia Wang[1], Ann Jeffers[1], Jake Boren[1], Shuzi Owens[1], Takahiro Suzuki[2], Masaaki Higashihara[2], Steven Idell[1], Torry A. Tucker[1] and Mitsuo Ikebe[1, 4]

[1] Department of Cellular and Molecular Biology, University of Texas Health Science Center Northeast, 11937 US Highway 271, Tyler, Texas, 75708-3154, USA

[2] Department of Hematology, Kitasato University School of Medicine, 1-15-1 Kitasato, Minami-ku, Sagamihara, Kanagawa 252-0374, Japan

[3] Equally contributed

To whom correspondence should be addressed. E-mail: Mitsuo.Ikebe@uthct.edu

Supplementary Information

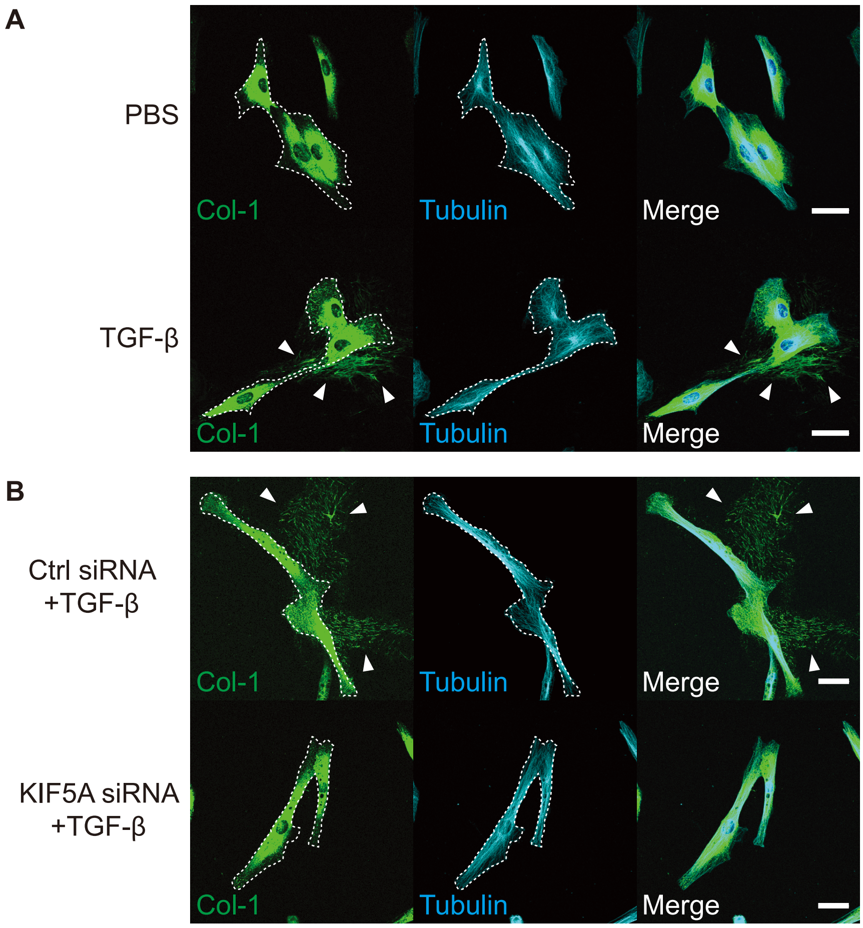

Supplementary Fig. S1. Immunofluorescent images of collagen secretion from HPMC. (A) Confocal images of collagen secretion in HPMCs with or without TGF-β stimulation. White dotted lines indicate the edge of cells. White arrowheads represent secreted collagen in outside of cells. Scale bar; 40 μm. (B) Effect of KIF5A KD on collagen secretion of HPMCs treated with control siRNA and KIF5A siRNA after TGF-β stimulation. White dotted lines indicate the edge of cells. White arrowheads represent secreted collagen in outside of cells. Scale bar; 40 μm.

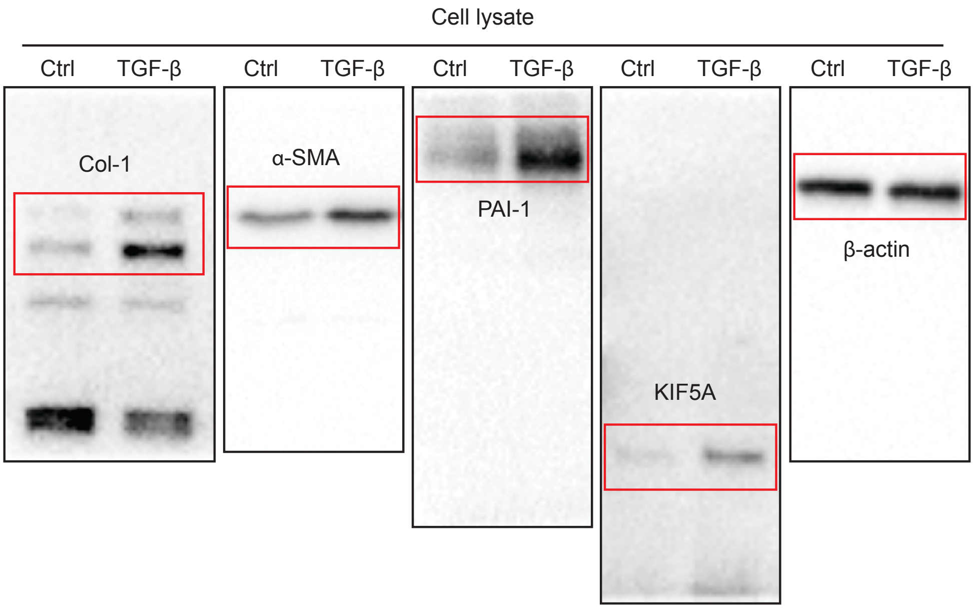

Supplementary Fig. S2. Full-length blots for Figure 1B.

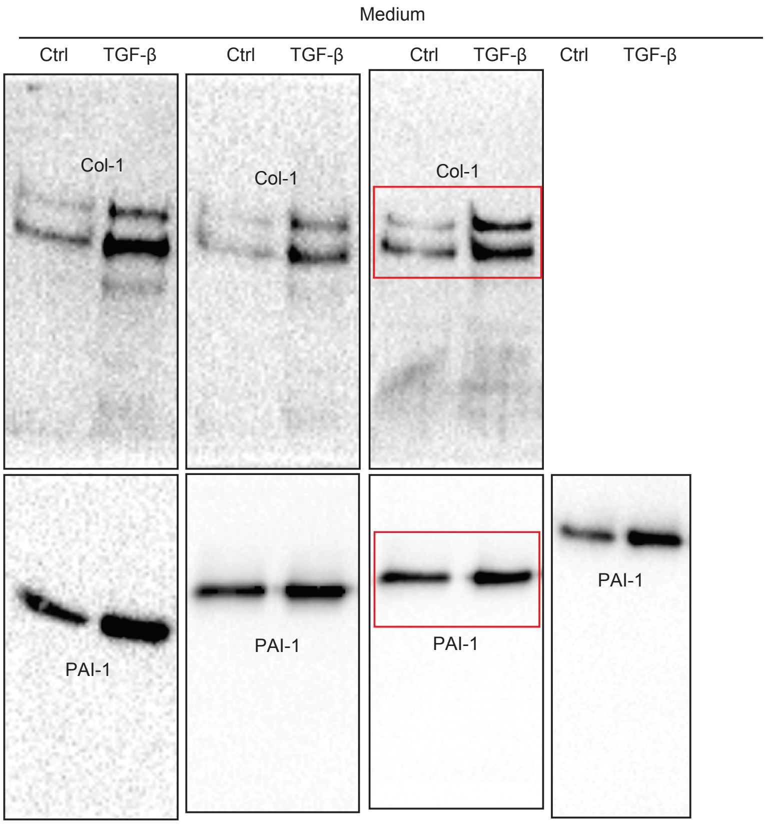

Supplementary Fig. S3. Full-length blots for Figure 1C.

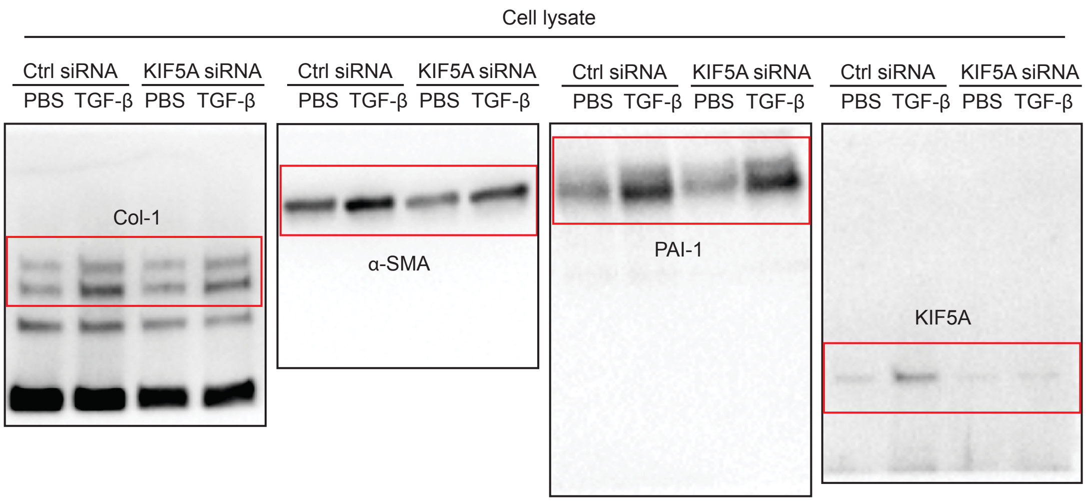

Supplementary Fig. S4. Full-length blots for Figure 4B.

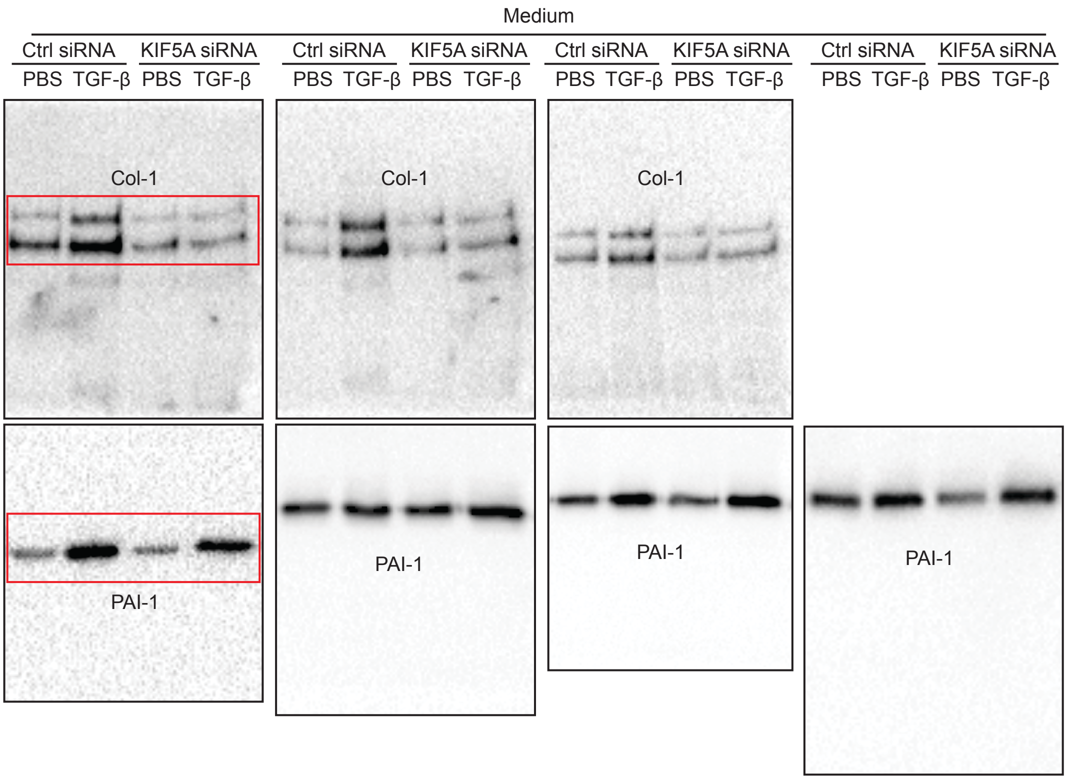

Supplementary Fig. S5. Full-length blots for Figure 4C.

Supplementary Video 1. Dual color live cell imaging of Col-1 (Green) and KIF5A (Red). Scale bar; 10 μm. Time stamp; min:sec.

Supplementary Video 2. Movements of filamentous Col-1 (Green). Scale bar; 10 μm. Time stamp; min:sec.
